# Ovarian cancer recurrence: is the definition of platinum sensitivity modified by PARPi, bevacizumab or other intervening treatments? : a clinical perspective

**DOI:** 10.20517/cdr.2022.01

**Published:** 2022-05-12

**Authors:** Peter G. Rose

**Affiliations:** Section of Gynecologic Oncology, Women’s Health Institute, Cleveland Clinic, Cleveland, Ohio 44195, USA.

**Keywords:** Maintenance, consolidation, PARP inhibitors, bevacizumab, radiation, chemotherapy

## Abstract

In view of the high risk of recurrent disease in stage III and IV ovarian cancer following primary first-line chemotherapy, a variety of maintenance and consolidation treatment strategies have been developed. These have included: radiation, intravenous or intraperitoneal chemotherapy, targeted therapies, and immunotherapy. Popular at this time is the use of Poly-adenosine ribose polymerase (PARP) inhibitors and bevacizumab as maintenance therapy. What effect these maintenance or consolidation therapies have on subsequent response to therapy, specifically platinum-based chemotherapy, is only beginning to be studied. In this manuscript, we review the impact of PARP inhibitors and bevacizumab as well as radiation and maintenance chemotherapy on subsequent response to treatment. Prior use of bevacizumab does not appear to adversely affect subsequent response to platinum-based chemotherapy or platinum-based chemotherapy with bevacizumab. Prior therapy with PARP inhibitors induces platinum resistance to subsequent platinum-based therapy and negates classic predictors of response such as platinum-free interval and breast cancer susceptibility gene (BRCA) mutational status.

## INTRODUCTION

Because of a lack of early detection methodology, most patients with ovarian cancer present with advanced-stage (stage III and IV) disease. Despite high complete response rates to surgery and primary chemotherapy, recurrence occurs in approximately 80% of these patients. Because of this very high recurrence rate, a variety of maintenance/consolidation strategies following completion of first-line chemotherapy have been tested in this patient population. Maintenance/consolidation strategies have included radiation, intravenous or intraperitoneal chemotherapy, targeted therapies, and immunotherapy^[1,2]^.

Despite maintenance/consolidation strategies, recurrence remains high. Little is known about the interaction of maintenance/consolidation therapies and their impact on subsequent response to treatment. In the era before maintenance therapy, once a patient recurs, a major factor in choosing a second-line therapy is the patient’s potential for retreatment with a platinum compound. In 1992, Markman and Hoskins were the first to recognize the importance of defining the recurrent ovarian cancer population and proposed the definition of platinum-sensitive and platinum-resistant disease^[3]^. Patients that progressed on therapy had less than a partial response or recurred within 6 months were considered platinum-resistant, while patients who had a previous partial or complete response and did not require chemotherapy for 6 months were considered potentially platinum-sensitive. Subsequent definitions have subdivided patients who recurred within 6 months to those who recur between 0 and 3 months as platinum-refractory *vs*. 3 to 6 months as platinum-resistant^[4]^. Additionally, platinum-sensitive patients have been subdivided into those who recur in 6 to 12 months as partially platinum-sensitive and those who recur more than 12 months as fully platinum sensitive. Other authors feel platinum sensitivity is a continuum without a strict time cut-off^[5]^. The duration of time off of platinum defines the platinum-free interval.

Platinum-containing drugs act mainly to induce DNA damage which activates a DNA damage response^[6]^. Tumors with deficiencies in homologous recombinant DNA repair are unable to repair platinum-induced DNA damage and undergo apoptosis. Approximately 20% of patients with ovarian cancer have a hereditary germline mutation in the Fanconi Anemia-BRCA pathway, which increases the risk of ovarian cancer and increases their response to chemotherapy^[7]^. An additional 30% of the patients with the most common type of ovarian cancer (high-grade serous carcinoma) may also have somatic mutations in the tumor, which increases the likelihood of responding to chemotherapy and are classified as homologous recombinant deficient (HRD)^[8]^. In addition to the platinum-free interval, the BRCA mutational and HRD status also is associated with improved response to second-line chemotherapy. Among patients with ovarian cancer patients with BRCA mutations, Tan *et al*.^[9]^ reported higher response rates (CR/PR) after second-line (70.6 *vs.* 38.7%; *P *= 0.035) and third-line (64.3 *vs.* 8.7%; *P *= 0.001) platinum-based chemotherapy compared to non-BRCA mutated patients.

Poly-adenosine ribose polymerase (PARP) enzymes play critical roles in the repair of single-strand DNA breaks (SSB) and maintenance of genomic integrity of a cell^[10]^. Like platinum compounds, inhibitors of poly-adenosine ribose polymerase (PARPis) have their greatest oncologic effect on tumors with germline BRCA mutations and HRD. However, even in *BRCA *mutated patients, response to PARP is closely related to the patient’s platinum sensitivity. Fong *et al.*^[11]^ demonstrated a variable clinical benefit rate across the platinum-sensitive, resistant, and refractory subgroups (69%, 45%, and 23%, respectively). This suggests a common mechanism of drug resistance. Ang *et al.*^[12] ^made the first report of the clinical outcome of post PARP inhibitor chemotherapy. Among 48 BRCA mutated patients who subsequently received post olaparib platinum-based chemotherapy, a response rate of 40% was noted. The authors suggested non-overlapping mechanisms of drug resistance. More recently, a number of authors have reported decreased response to second- and third-line platinum-based chemotherapy in BRCA mutated patients following PARPi therapy Table 1. Cecere *et al. *^[13] ^studied 234 BRCA mutated patients that received olaparib after at least two platinum-based regimens. Eighty-eight percent of these patients received 2-4 platinum-based regimens. Sixty-six of these patients received post olaparib chemotherapy. These patients were stratified by their platinum-free interval. Patients with platinum-free intervals of greater than 12 months predominantly (78%) received a platinum regimen. Fifty-two percent of patients with platinum-free intervals of 6-12 months received a platinum regimen. No patients with platinum-free intervals of < 6 months received a platinum regimen. The response rates were low for all platinum-free intervals. Baert *et al.*^[14]^ evaluated the rate of disease progression to third-line platinum chemotherapy among 35 BRCA mutated and non-mutated patients. Prior PARP inhibitor treatment significantly increased the frequency of disease progression by 40% *vs*. 9% in non-PARP inhibitor-treated controls. Frenel *et al.*^[15] ^evaluated the progression-free survival following third-line platinum-based chemotherapy among BRCA mutated patients enrolled in SOLO-2. The progression-free survival to third-line platinum-based chemotherapy was significantly reduced in the olaparib-treated patients by 7 *vs*. 14.3 months in the placebo-treated controls. Rose *et al.*^[16] ^studied BRCA mutated patients who received second- or third-line platinum-based chemotherapy. Patients who received a PARP inhibitor following first- or second-line chemotherapy had a poorer progression-free survival to second- or third-line platinum-based chemotherapy, respectively (8 months *vs.* 19.1 months). This study also demonstrated that there was no prognostic significance to the platinum-free interval as the progression-free survival among BRCA mutated patients with prior platinum-free intervals of 6 to 12 months, 12 to 24 months, or more than 24 months was the same. Additionally, there was no prognostic significance to BRCA mutational status as the progression-free survival of patients to subsequent platinum-based chemotherapy who were BRCA mutated or non-BRCA mutated following prior PARP inhibitor exposure was the same.

**Table 1:** Studies of chemotherapy following PARP inhibitor therapy

**Author Year**	**Study design**	**Patients **	**Results**
Ang* et al.*^[12]^ 2013	retrospective	Post olaparib platinum-based chemotherapy among BRCA mutated	48 patients RR 40%
Cecere *et al*.^[13]^ 2020	retrospective	Post olaparib chemotherapy* among BRCA mutated	> 12 mo 14 platinum, 4 non-platinum RR 22% 6-12 mo 14 platinum, 13 non-platinum RR 11%
Baert* et al.*^[14]^ 2020	retrospective	PD on 3^rd^ platinum-based among BRCA mutated and nonmutated	No prior PARP (*n *= 57): PD 9% *P *= 0.003 Prior PARP (*n *= 35): PD 40%
Frenel *et al.*^[15] ^2020	SOLO-2	PFS following 3^rd^ platinum-based chemotherapy among BRCA mutated	Olaparib (*n *= 54): 7 months Placebo (*n *= 42): 14.3 months
Rose *et al.*^[16]^ 2021	retrospective	PFS following 2^nd^ & 3^rd^ platinum among BRCA mutated	2^nd^ platinum No prior PARP (*n *= 108) 19.1 mo *P *= 0.005 Prior PARPi (*n *= 7) 8.0 mo 3^rd^ platinum No prior PARP (*n *= 42) 18.4 mo *P *< 0.001 Prior PARPi (*n *= 13) 7.9 mo 2^nd^ or 3^rd^ platinum No prior PARP (*n *= 150) 19.1 mo *P *< 0.001 Prior PARPi (*n *= 20) 8.0 mo

PD: progressive disease; PFS: progression-free survival; *not able to associate platinum and response; PARP: poly-adenosine ribose polymerase.

These findings may explain why PARP inhibitors demonstrate significant improvements in progression-free survival but not overall survival. While the mechanisms of platinum and PARP inhibitor resistance have only begun to be determined, Johnson *et al.*^[17]^ did demonstrate stabilization of mutant *BRCA*1 protein confer PARP inhibitor and platinum resistance.

Another unresolved issue regarding PARP inhibitor utilization is when PARP inhibition therapy should be instituted. In study 19, which studied olaparib maintenance after second-line platinum-based chemotherapy, a significant improvement in overall survival was only evident when placebo-treated patients who received subsequent PARP inhibitor therapy were excluded from the analysis^[18]^. This implies that delayed PARP inhibition may be just as effective as immediate PARP inhibition. The conclusion of the SOLO-2 analysis abstract by Frenel *et al.*^[15]^ states, “the earlier use of olaparib remains optimal”. The basis for this conclusion is uncertain. The marginal improvement in overall survival in SOLO-2 (upper limit of 95% confidence interval 1.00) is likely due to the fact only 38% of the placebo-treated patients were able to receive a PARP inhibitor^[19]^. More recently, the lack of overall survival benefit with niraparib in the Nova trial, which studied niraparib as second-line maintenance in BRCA mutated, HRD positive and HRD negative patients questions the importance of early PARP inhibitor therapy^[20]^. Although specific post clinical trial treatment was not collected in this study, it is postulated that many of the placebo-treated control patients subsequently received a PARP inhibitor because of their current greater availability commercially.

This emphasizes the need for a prospective randomized trial to compare the early use of a PARP inhibitor to the delayed use of a PARP inhibitor. In the design of this trial, one would have to think about when early PARP inhibitor therapy should occur. Numerous studies are suggested that 30% of BRCA mutated patients may not recur^[21-23]^. Therefore, is it reasonable from a quality of life, toxicity and cost perspectives to treat 100% of the BRCA mutated patients after first-line chemotherapy when only 70% of the patients may recur? Alternatively, would it be better to delay maintenance therapy once a patient has developed a recurrence?

As reported by Burger *et al.*^[24]^, “Vascular endothelial growth factor and angiogenesis correlate directly with the extent of disease and inversely with progression-free survival and overall survival.” Several antiangiogenic targeted therapies have been studied in ovarian cancer. The most widely studied antiangiogenic agent is bevacizumab. Two studies conducted in Europe (ICON 7) and in the United States Gynecologic Oncology Group (GOG) 218 demonstrated a benefit of maintenance bevacizumab in stage IV patients^[25,24]^. In ICON 7, patients were defined as high risk for progression, which included stage IV disease, inoperable stage III disease, or suboptimally debulked (> 1 cm) stage III disease. The addition of bevacizumab to chemotherapy and as maintenance for 12 cycles resulted in an improvement in overall survival [39.7 months *vs.* 30.2 months (HR 0.78, *P *= 0.03)]^[25]^. For stage IV patients in GOG 218, the addition of bevacizumab to chemotherapy and maintenance improved the median survival by 10 months (42.8 months *vs*. 32.6 months, HR 0.75)^[26]^. However, no statistical benefit was seen in an exploratory analysis using ICON 7 criteria, suboptimal stage III and IV patients *vs*. all other stage III patients with survivals of 42.8 months *vs*. 45.6 months, respectively. The benefit of bevacizumab in the ICON 7 high risk for progression subgroup included inoperable stage III patients treated with neoadjuvant chemotherapy^[25]^. GOG 218 did not include patients treated with neoadjuvant chemotherapy and therefore cannot confirm or refute ICON 7 findings in this population.

Whether prior exposure to bevacizumab affects the response to subsequent platinum-based chemotherapy or platinum-based chemotherapy with bevacizumab has been evaluated in three randomized trials. In GO G 213, 10% of patients in each arm had received bevacizumab^[27]^. Despite prior bevacizumab, patients who were randomized to retreatment with bevacizumab had an improved progression-free survival, but this was not statistically significant as they comprised only 10% of the study population [HR 0.545 (0.292-1.017)]. In contrast, in the MITO16b/MANGO-OV2/ENGOT-ov17 trial, patients who received platinum-based chemotherapy with bevacizumab as part of first-line therapy were randomized to receive a platinum-based chemotherapy doublet either carboplatin and paclitaxel, carboplatin and gemcitabine or carboplatin and pegylated liposomal doxorubicin, with or without bevacizumab^[28]^. The median progression-free survival to second-line platinum-based chemotherapy doublet was 8.8 months. The response rate for second-line platinum-based chemotherapy was dependent on the platinum-free interval, with different response rates for patients who progress at 6 to 12 months *vs*. those who progress after 12 months^[4]^. Table 2 is a list of platinum-based second-line chemotherapy regimens based on an increasing percentage of patients in the 6- to 12-month interval. GOG 213^[27]^ and ICON 4^[29]^ have a more favorable population of patients with a lower frequency of 6- to 12-month platinum-free interval patients 25% and 27%, respectively, compared to MITO16b/MANGO-OV2/ENGOT-ov17 with 35%. The MITO16b/MANGO-OV2/ENGOT-ov17 trial median progression-free survival of 8.8 months compares favorably to other platinum doublets in platinum-sensitive recurrent ovarian cancer. The median progression-free survival to second-line platinum-based chemotherapy doublet was not statistically different at 9.6 and 8.0 months, for patients who had progressed after bevacizumab or progressed on bevacizumab, respectively *P *= 0.8.

**Table 2:** Chemotherapy only arms from randomized trials of platinum-based chemotherapy in platinum-sensitive ovarian cancer

**Study** **Invest Agent** **Regimen**	**GOG 213^[27]^** **Bev** **P C**	**ICON 4^[29]^** **None** **P C**	**MME^[28]^** **Bev** **P C**	**Calypso^[30]^** **None** **PLD C**	**Calypso^[30]^** **None** **P C**	02004093*^[31]^ **Pertuzumab** **P C & G C**	**AGO 2.2^[32]^** **None** **G C**	**Oceans^[33]^** **Bev** **G C**	00318370*^[34]^ **Farletuzumab** **P C**	00170677*^[35]^ **None** **T C**
6-12 mo	25%	27%	35%	35%	36.1%	38.7%	39.9%	42.1%	53.3%	64%
> 12 mo	75%	73%	65%	65%	63.9%	61.3%	59.8%	57.9%	46.7%	36%
PFS	10.4 mo	12 mo	8.8 mo	11.3 mo	9.4 mo	8.7 mo	8.6 mo	8.7 mo	9.0 mo	10 mo

NCT number*; Bev: bevacizumab; C: carboplatin; G: gemcitabine; MME: MITO16b/MANGO-OV2/ENGOT-ov17; P: paclitaxel; PLD: pegylated liposomal doxorubicin; T: topotecan; GOG: Gynecologic Oncology Group.

The AGO OVAR 2.21/NGOT- ov 18 trial, which compared carboplatin/gemcitabine/bevacizumab with carboplatin/pegylated liposomal doxorubicin/bevacizumab, allowed prior antiangiogenic therapy^[36]^. Prior antiangiogenic therapy was received in 47.5% of all patients, most commonly bevacizumab (87.3%). Prior antiangiogenic therapy did statistically decrease the median progression-free survival at 10.1 months (95%CI: 8.5-11.2) *vs*. 13.6 months (95%CI: 11.7-15.6) for carboplatin/gemcitabine/bevacizumab but not for carboplatin/pegylated liposomal doxorubicin/bevacizumab 11.3 months (95%CI: 10.1-13.8) *vs*. 14.4 months (95%CI: 12.3-16.8). The median overall survival was decreased more than 5 months with prior antiangiogenic therapy 25.1 months (95%CI: 19.3-27.8) *vs*. 30.7 months (95%CI: 27.4-36.2) for the carboplatin/gemcitabine/bevacizumab arm and by almost 10 months, 27.7 months (95%CI: 24.0-31.7) *vs*. 37.3 months (95%CI: 30.9-42.9) for the carboplatin/pegylated liposomal doxorubicin/bevacizumab arm, but these were not statistically significant.

Thirty-one percent of patients in each arm were in the 6-12 months platinum-free interval, so this progression-free survival is consistent with prior studies Table 2 and does not suggest any negative impact of prior antiangiogenic therapy.

## RADIATION CONSOLIDATION

Ovarian cancer is radiosensitive, but the potential for metastasis throughout the abdominal cavity requires whole abdominal radiation. While bulky residual disease is difficult to treat, it was believed that patients who had undergone cytoreductive surgery and platinum-based chemotherapy, with the potential for further chemical cytoreduction, consolidation with whole abdominal radiation might be more effective. Lawton *et al.*^[37]^ from the West Midlands Ovarian Cancer Group randomized patients to whole abdominal radiation therapy using a moving strip technique or one year of chlorambucil. Overall survival at two years was 35%, with no significant difference in survival between the two groups despite the fact that approximately 50% of the patients were optimally debulked prior to consolidation. Toxicity was considerable in both arms, and almost 50% of patients were unable to complete the planned treatment in both arms. A subsequent British trial by the North Thames Group evaluated consolidation platinum-based chemotherapy *vs*. whole abdominal radiation^[38]^. One hundred seventeen patients with residual disease of 2 cm or less at second-look laparotomy or laparoscopy were then randomized to receive consolidation therapy, either five further courses of carboplatin at the same dosage or whole-abdominal RT (24 Gy). The median survival for the whole group from the date of surgery was 25 months. No statistical difference was found in either survival or disease-free survival between those patients who received consolidation chemotherapy and those who were treated with abdominal RT. The Swedish-Norgewian Ovarian Cancer Study Group performed an observation controlled trial comparing receive whole-abdominal irradiation (WAR) or six courses of consolidation chemotherapy (cisplatin 50mg/m^2^ and doxorubicin 50mg/m^2^ or epirubicin 60mg/m^2^) *vs*. no further treatment^[39]^. Seven hundred and 42 patients were prospectively studied. Following primary cytoreductive surgery followed by induction chemotherapy (four courses of cisplatin 50mg/m^2^ and doxorubicin 50mg/m^2^ or epirubicin 60mg/m^2^), patients with no evidence of disease (NED), a complete clinical response (cCR), or partial response (cPR) underwent a second-look laparotomy. At second-look laparotomy, patients were classified as having a pathologic complete response, microscopic residual disease, or macroscopic residual disease. One hundred and seventy-two patients with a pathologic complete response or microscopic residual disease entered into the randomized study. Patients with a pathologic complete response who received radiation therapy had improved progression-free survival compared to patients who received chemotherapy or observation (*P *= 0.032), but overall survival was not improved (*P *= 0.084). Subsequent post-study treatment was not collected or reported in this trial. Preclinical data suggests a possible interaction between radiation resistance and chemotherapy resistance^[40]^. Since whole abdominal radiation is not widely utilized clinically, a clinical correlation has not been documented in the literature.

Lastly, although Intraperitoneal P32 was not found to be an effective consolidation therapy for stage III ovarian cancer patients after negative second-look laparotomy, it did not adversely affect overall survival^[41]^.

## MAINTENANCE CHEMOTHERAPY

In 2003, Markman reported the results of the SWOG/GOG 178 trial, which demonstrated that following first-line chemotherapy, patients randomized to 12 cycles of paclitaxel *vs*. three cycles of paclitaxel had an improved progression-free survival (28 months *vs.* 21 months *P* < 0.005)^[42]^. Once this progression-free survival improvement was reported, patients in the 3 cycle arm were offered additional therapy. Possibly, as a result of this delayed crossover, there was no difference in overall survival. Conte *et al.*^[43]^ performed a trial of six cycles of paclitaxel *vs*. observation following primary chemotherapy. No difference in overall survival was noted. The Gynecological Oncology Group performed a larger confirmatory trial (GOG 212) comparing 12 cycles of paclitaxel *vs*. 12 cycles of polyglutamate paclitaxel (Xyotax) *vs*. observation^[44]^. No difference in overall survival was noted at 51.3 months, 60.0 months, and 54.8 months for paclitaxel, Xyotax, and observation, respectively. Other studies have used topotecan^[45,46]^ or epirubicin^[47]^ as maintenance therapy *vs*. observation, but no difference in overall survival has been noted. In summary, although multiple chemotherapy agents have been studied as maintenance therapy and ovarian cancer, it is clear that they have not improved overall survival. When non-platinum maintenance chemotherapy was utilized, since patients were off platinum, the platinum-free interval was not affected, and anecdotally subsequent chemotherapy was not adversely affected by non-platinum maintenance chemotherapy.

In summary, patients with advanced-stage ovarian cancer have high responses to primary therapy but have a high recurrence rate. Multiple studies have looked at the use of maintenance or consolidation therapy to prevent recurrence. Currently, both the use of bevacizumab and PARP inhibitors are favored as possible maintenance therapies. Prior use of bevacizumab does not appear to adversely affect subsequent response to platinum-based chemotherapy or platinum-based chemotherapy with bevacizumab. Since the response to PARP inhibitors is related to platinum sensitivity, it suggests a common mechanism of resistance. Prior therapy with PARP inhibitors induces platinum resistance to subsequent platinum-based therapy and negates classic predictors of response such as platinum-free interval and BRCA mutational status. Randomized trials are needed to determine the best time to initiate PARP inhibitor therapy.

## DECLARATIONS

### Copyright

© The Author(s) 2022.
